# Design of a Low-Power, Small-Area AEC-Q100-Compliant SENT Transmitter in Signal Conditioning IC for Automotive Pressure and Temperature Complex Sensors in 180 Nm CMOS Technology

**DOI:** 10.3390/s18051555

**Published:** 2018-05-14

**Authors:** Imran Ali, Behnam Samadpoor Rikhan, Dong-Gyu Kim, Dong-Soo Lee, Muhammad Riaz Ur Rehman, Hamed Abbasizadeh, Muhammad Asif, Minjae Lee, Keum Cheol Hwang, Youngoo Yang, Kang-Yoon Lee

**Affiliations:** 1 College of Information and Communication Engineering, Sungkyunkwan University (SKKU), Suwon 16419, Korea; imran.ali@skku.edu (I.A.); behnam@skku.edu (B.S.R.); rlarlarbrb@skku.edu (D.-G.K.); blacklds@skku.edu (D.-S.L.); riaz@skku.edu (M.R.U.R.); hamed@skku.edu (H.A.); m.asif@skku.edu (M.A.); khwang@skku.edu (K.C.H.); yang09@skku.edu (Y.Y.); 2 School of Information and Communications, Gwangju Institute of Science and Technology (GIST), Gwangju 61005, Korea; minjae@gist.ac.kr

**Keywords:** AEC-Q100, automotive, CMOS, low-power, pressure sensor, SAE J2716, single edge nibble transmission (SENT), signal conditioning IC, temperature sensor

## Abstract

In this paper, a low-power and small-area Single Edge Nibble Transmission (SENT) transmitter design is proposed for automotive pressure and temperature complex sensor applications. To reduce the cost and size of the hardware, the pressure and temperature information is processed with a single integrated circuit (IC) and transmitted at the same time to the electronic control unit (ECU) through SENT. Due to its digital nature, it is immune to noise, has reduced sensitivity to electromagnetic interference (EMI), and generates low EMI. It requires only one PAD for its connectivity with ECU, and thus reduces the pin requirements, simplifies the connectivity, and minimizes the printed circuit board (PCB) complexity. The design is fully synthesizable, and independent of technology. The finite state machine-based approach is employed for area efficient implementation, and to translate the proposed architecture into hardware. The IC is fabricated in 1P6M 180 nm CMOS process with an area of (116 μm × 116 μm) and 4.314 K gates. The current consumption is 50 μA from a 1.8 V supply with a total 90 μW power. For compliance with AEC-Q100 for automotive reliability, a reverse and over voltage protection circuit is also implemented with human body model (HBM) electro-static discharge (ESD) of +6 kV, reverse voltage of −16 V to 0 V, over voltage of 8.2 V to 16 V, and fabricated area of 330 μm × 680 μm. The extensive testing, measurement, and simulation results prove that the design is fully compliant with SAE J2716 standard.

## 1. Introduction

With rapid improvement in technology, the utilization of complex electronic systems has increased in automotive applications for comfort, safety, and efficiency. Electronic devices are rapidly taking the place of mechanical components in automotive industry. Today’s research focuses on reducing analog circuit complexity and on increasing the amount of digital signal processing to minimize cost, size, and power consumption, with enhanced reliability and improved efficiency. Modern automobiles are now equipped with complex electromechanical systems comprised of dozens of inter-networked ECU devices and sensors [1]. Among others, the most critical components of these automotive electronic control systems are the sensors and actuators that convey physical quantities to ECU in the form of electrical signals [2]. The micro-electro-mechanical systems (MEMS) sensors are attaining great attention in a variety of applications including automotive, IoT, medical, agricultural, industrial, etc. [3,4,5,6]. The controller area network (CAN) and local interconnect network (LIN) are typical in-vehicle networks to connect ECU and actuators [7].

In the present day automotive applications, over 100 sensors per vehicle are fitted to minimize human inattention and mistakes, fulfill environmental rules and regulations and to enhance traffic flow [8]. These sensors are being used for measuring pressure, temperature, position, angular rate, acceleration, comfort factors, etc. [9]. Until now, analog signals and techniques are the main source of communication between the ECU and actuators [10]. Different methods have been reported in the literature for conveying the sensor data to processing units. In [11], a compressed sensing AFE for bio-sensor uses DAC at the output interface for transmitting sensor data. The interface is an analog single line, and DAC takes a huge IC area, consumes relatively more power, and is sensitive to noise. Reference [12] focuses on a sensor interface IC for biomedical applications, in which the ADC outputs the sensor data. In this case, multiple pins are required for the sensor interface, which demand more IC PADs, increase IC area and cost, and make connectivity and PCB more complex. Reference [13] presents a temperature sensor with high-resolution first order sigma-delta modulator (SDM). This design provides a single line digital stream. It requires additional filtering modules before retrieving the sensor information in the ECU, which is additional cost. For automotive applications, a capacitive sensor AFE is discussed in [4], in which addition processing modules, such as analog-to-digital converter (ADC), gain and offset register banks, and serial interface are used outside the IC. Reference [14] incorporates a digital serial interface into the AFE for electrocardiogram (ECG) and electroencephalogram (EEG) monitoring applications, which requires three interface signals for communication with the external processing unit. Reference [15] discusses a signal conditioning and calibration method for sensors for an automotive tire pressure monitoring system (TPMS), which incorporates wireless sensors, and a high-frequency antenna for transmitting sensor data to the central receiver. For the high-resolution sensors, such as manifold air pressure sensors, mass air flow sensors and throttle position sensors [16], conventional techniques and protocols are no longer suitable for reporting the sensor information to the ECU. In the automotive industry, SENT, a SAE J2716 digital communication protocol is the emerging technique for high-resolution data transmission for automotive applications [17,18,19,20].

The focus of this paper is a highly reliable low-power, small-area SENT transmitter architecture with protection. It includes the detailed SENT architecture, reverse voltage and over voltage protection circuit for automotive reliability, and IC microphotograph and measurement results. The proposed design is integrated with a pressure and temperature complex sensor signal conditioning IC for automotive applications. This design has the strength to be incorporated into any sensor for communicating with the ECU in the automotive. It is a very simple digital architecture that offers simple connectivity, low cost, smaller area, and immunity to noise. For automotive reliability, the reverse and over protection circuit [21] makes it compliant with AEC-100Q [22]. After gain adjustment, amplification, analog-to-digital conversion and essential processing on sensor signal, it is applied to the input of SENT block for transmission. The SENT transmitter encodes data according to SAE J2716, and hands it over to the ECU for monitoring and action.

The rest of the paper is organized as follows: Section 2 briefly reviews the SENT protocol. Section 3 covers the block level structure of the pressure and temperature sensor frond end in which the proposed SENT design is integrated. Section 4 investigates the detailed architecture of the proposed design for the SENT transmitter. Section 5 discusses the reverse voltage and over voltage protection circuit. Section 6 summarizes the measurement and simulation results, while Section 7 concludes the paper.

## 2. Overview of the SENT Protocol

SENT protocol is the emerging technique in the automotive industry. This encoding scheme is adopted for communicating the high-resolution sensor information from the sensor to the ECU. The SENT digital communication protocol is anticipated as a replacement for the lower resolution ADC and pulse width modulation (PWM) methods. It is a promising low-cost solution [23], and a smart alternative to the controller area network (LIN) and local interconnect network (CAN) [17]. SENT is a unidirectional communication scheme from the sensor to the controller and does not require any synchronization or control signals from the receiver module. The ECU provides the supply voltage and ground reference to the transmitter module. In response, the SENT transmitter provides a digitally encoded stream to the ECU receiver module through a single signal wire. Therefore, at the physical layer, only three connection wires are required between the transmitter and receiver modules for this protocol.

**Figure 1:**
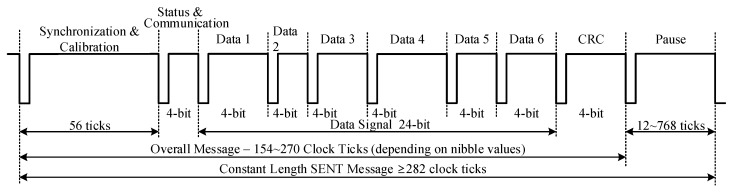
SENT message sequence.

**Figure 2:**
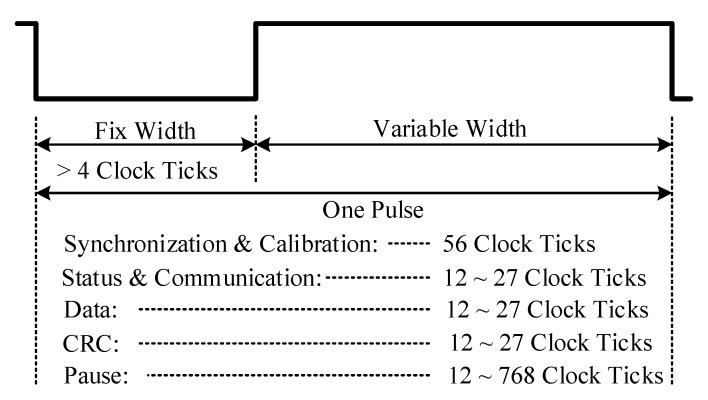
SENT pulse timing characteristics.

After encoding through this protocol, the sensor signal is transmitted as a series of variable width pulses with data encoded as falling to falling edge periods. The message pulse order is predefined for the transmitter. Figure 1 shows the framing structure of a SENT message sequence. There are five variants of pulses in one message sequence. The Synchronization and Calibration pulse is followed by the Status and Communication, Data, Cyclic Redundancy Check (CRC), and Pause pulses. Each pulse remains low for the fixed number of clock ticks. The pulse remains high for the variable duration, which depends upon the type of pulse and nibble value. Figure 2 summarizes the timing characteristics of each pulse. The transmission clock period for this protocol is from 3~90 μs. The Synchronization and Calibration pulse indicates the start of the message sequence. Its period has a fixed duration of 56 clock ticks. It facilitates the ECU for synchronization of the frame stream, and the recovery of clock and data information. The Status and Communication nibble pulse enables the transmitter to report sensor-related miscellaneous information, such as part number, version, error codes, etc., to the ECU. The 4-bit Data pulses encapsulate the value of the signal to be communicated, and the number of these pulses may range from one to six. The number of nibbles is fixed for each application but may vary among different applications. The CRC pulse is the checksum information and is calculated from data nibbles of each message sequence. The Pause pulse is an optional fill pulse in SENT transmission after CRC nibble. It maintains the constant number of clock ticks for each message sequence and provides a relief for ECU to process the current frame. The SENT protocol supports two modes for communication, namely fast channel communication, and slow channel communication. In fast channel mode, the signal information is transmitted as one to six data nibbles. The short serial message format and enhanced serial message format are used in slow channel mode. In both slow channel formats, specific sensor related information is transmitted to ECU in Status and Communication nibbles in several consecutive message sequences.

## 3. Architecture of the Signal Conditioning IC for Pressure and Temperature Sensors

**Figure 3:**
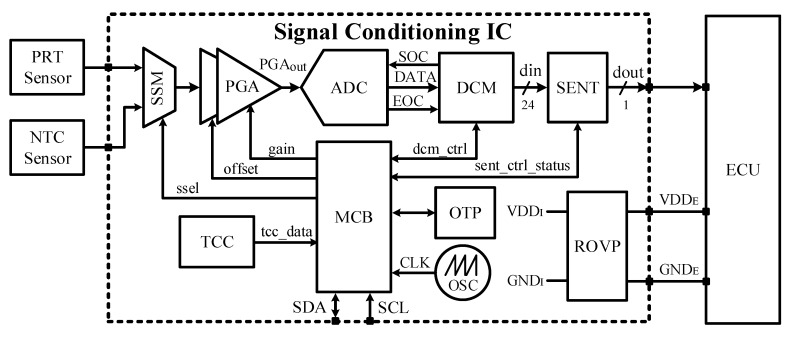
Signal conditioning IC for pressure and temperature sensors.

Figure 3 shows a block diagram of the front-end, housing the proposed SENT transmitter design. It takes the pressure and temperature values from piezo-resistive type (PRT) and negative temperature coefficient (NTC) sensors, respectively, processes for gain and offset compensations, converts to digital bits, and encodes according to SENT protocol after calibration. The PRT Sensor uses the phenomenon of piezo-resistivity, in which the electrical resistance of a material changes in response to mechanical pressure and stress. The metal is a kind of piezo-resistive material to some extent, but nowadays, pressure sensors utilize semiconductor silicon as piezo-resistive material. The four piezo-resistors are connected electrically in a Wheatstone bridge configuration. When mechanical pressure is applied on its surface, the output voltage changes. Due to the smaller size, direct signal transduction mechanism, easy integration, etc., piezo-resistive pressure sensors have been most commonly used in the automobile, aerospace, and petrochemical fields for pressure measurements [24]. The NTC Sensor is a non-linear type and is made of semiconductor material whose electrical resistance decreases rapidly with small increase in temperature. Other than PRT and NTC sensors, the major building blocks for this processing IC include the programmable gain array (PGA), ADC, digital calibration module (DCM), main controller block (MCB), SENT transmitter (SENT), and reverse and over voltage protection (ROVP) circuit. The multiplexer (MUX) selects one sensor at a time and passes its measured value to the PGA. The same signal path is used for processing both temperature and pressure sensor data after MUX. The PGA analog block is responsible for gain and offset compensation in the input sensor signal, as the sensors have different sensitivity and offset values. It amplifies the voltage obtained from the sensors and enhances the resolution ability. After compensation, the PGA output has the same range, regardless of the sensors. It also ensures the constant polarity of the output signal, irrespective of the polarity of input signal. The ADC has 10-bit resolution with dual sample successive approximation (SAR) type architecture. It takes the analog PGA output, and converts it into 10-bit digital data. The DCM controls the ADC operation, compensates the linearity of the sensors, and increases the resolution of the input signals. It calibrates the IC for the minimum and maximum applied pressure and temperature ranges. After the calibration, the final parameters, such as the PGA gain, offset, and linearity compensation, are stored in one-time programmable (OTP) read only memory (ROM). The DCM output is 24-bit, in which the 10 least significant bits represent the temperature value, while the remaining 14 most significant bits are pressure sensor data. The MCB monitors and controls each activity of the signal conditioning IC. It facilitates the calibration process, alternately selects the PRT and NTC sensors, and adjusts the PGA gain and offset. An inter-integrated circuit (I2C) slave controller is also embedded into MCB for its communication with the external environment during the calibration process. The oscillator (OSC) is a spread spectrum clock source, and it generates the triangular profile signal from self-clocked feedback. It controls the frequency of the relaxation oscillator. Moreover, its frequency is also controllable from MCB. The effect of temperature variations on the PRT and NTC sensors are canceled by the temperature compensation circuit (TCC). The final digital code from MCB after all specified processing is handed over to SENT, which encodes it according to SAE J2716 standard.

## 4. The Architecture of the SENT Transmitter

**Figure 4:**
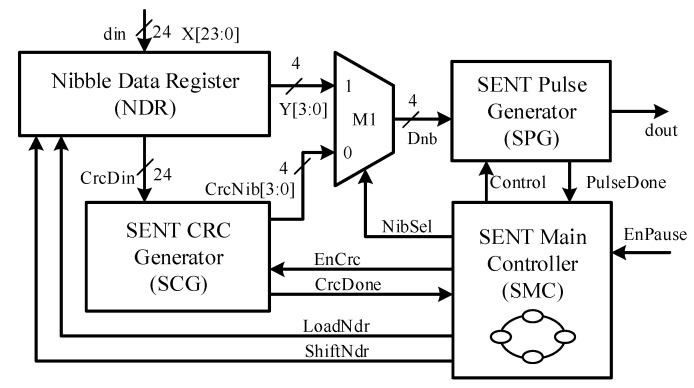
Proposed SENT transmitter architecture.

Figure 4 shows an architectural view of the proposed SENT design, which is hardware friendly. The design is primarily partitioned into the SENT main controller (SMC), SENT CRC generator (SCG), SENT pulse generator (SPG), and nibble data register (NDR) sub-blocks, based on their functionality.

The number of clock ticks, *N_p_* of each pulse has *N_low_* and *N_high_* count values, representing the low and high duration, and is given in Equation (1):

(1) $$N_{P\;}=\;N_{low}+N_{high}$$

According to SAE J2716, the *N_low_* must be greater than four, and it is fixed for all types of pulses in the message sequence for a particular application. The *N_high_* is variable, and its value depends on the type of pulse. The number of clock cycles, *N_nb_* of each nibble pulse (Status and Communication, Data and CRC) is described in Equation (2):

(2) $$N_{nb}=\;K_{offset}+D_{nb}$$

where the *K_offset_* is 12, and *D_nb_* is the value of nibble, which may be from 0 to 15. Hence, *N_nb_* ranges from (12 to 27) clock ticks, as the nibble value *D_nb_* changes from (0 to 15), respectively. The total clock cycles for nibble pulses *N_tnb_* is accumulated using Equation (3), as follows:

(3) $$N_{tnb}=\;N_{scn}+\overset{k}{\underset{i=0}{\sum }}N_{dn,i}+D_{nb}$$

where *k* is the total number of Data nibbles, and *N_scn_* and *N_cn_* are the number of clock ticks for Status and Communication and CRC nibbles, respectively. Equation (4) calculates the total number of clock ticks, *N_ms_* of one message sequence, in which *N_scp_* and *N_pp_* are the clock counts for Synchronization and Calibration and Pause pulses, respectively:

(4) $$N_{tms}=\;N_{scp}+N_{tnp}+N_{pp}$$

The total duration *T_ms_* of each frame is obtained by multiplying the *N_ms_* and clock period *T_clk_*, given in Equation (5):

(5) $$T_{ms}=\;T_{clk}\times N_{tnp}=\;\frac{N_{ms}}{f_{clk}}$$

When the Pause pulse is activated, the *T_ms_* is fixed for all message frames. In this case, the *N_pp_* varies in each message frame, to make the total length constant. The architectural detail of each part is described in the following subsections.

### 4.1. SENT Main Controller (SMC)

**Figure 5:**
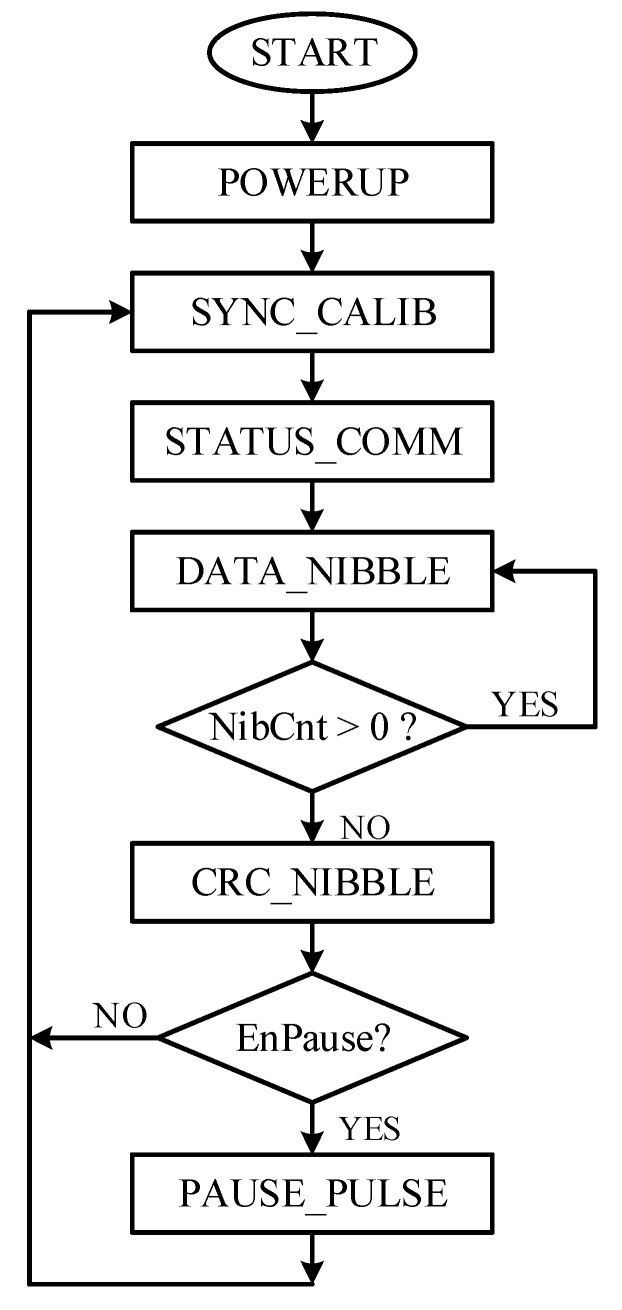
SENT main controller flow chart.

**Figure 6:**
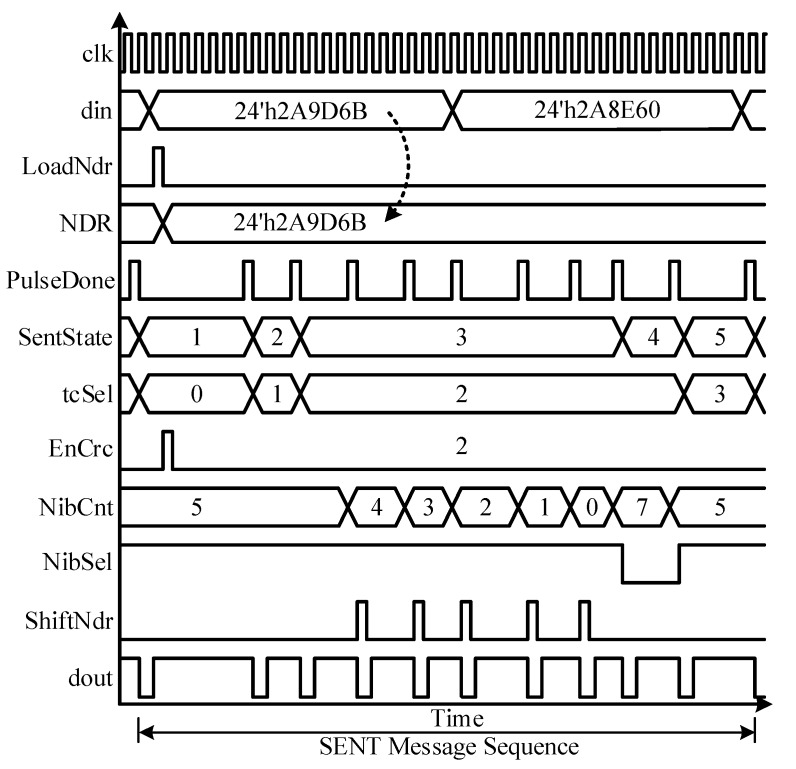
SENT main controller timing.

The SENT Main Controller is the brain of the proposed design and is based on the finite state machine (FSM) model. It supervises other blocks, to encode the incoming sensor information from DCM into SAE J2716 compliant message sequences. It generates the control signals, observes the status responses, and manages the sequence of each pulse generation. Figure 5 shows the flowchart of the SMC. Figure 6 shows the conceptual timing diagram that elaborates the related control and status signals for the controller itself, and the entire datapath, including NDR, SCG, SPG, and M1 modules. The controller, SMC enters into POWERUP state, after reset or start-up condition. It remains in this state for only one clock cycle. All the registers and control signals settle to their default values, and the dout signal is pulled up during this state. After power-up, the controller jumps to the SYNC_CALIB state. The Synchronization and Calibration pulse is generated in this state. The pulse is low for N_low_ clock cycles and remains high to complete N_scp_ clock ticks. When the controller enters into this state, the LoadNdr signal is asserted high for one clock cycle to sample din, 24-bit sensor information to NDR. These six nibbles are also routed to SCG block, and it is triggered to compute the checksum for the current message sequence. The CRC nibble value is calculated in eight clock cycles during this state. The controller state register, SentState is incremented for transmitting Status & Communication pulse in the next STATUS_COMM state. In this design, only the fast channel is used, and the optional slow channel is disabled. Therefore, the controller remains in this state for the minimum possible *K_offset_* clock ticks and enters into the DATA_NIBBLE state. All the six nibbles are encoded in this state. The *NibSel* remains high, and ShiftNdr toggles after every data nibble encoding to shift the NDR four bits left and load the next nibble value to the SPG. The counter NibCnt facilitates the controller to track the number of transmitted data nibbles. After all the six data nibbles are encoded, the next state in the queue is CRC_NIBBLE, in which the checksum nibble, manipulated from SCG for the current message frame, is transmitted. For this, the NibSel is deasserted low, to convey the computed CRC nibble CrcNib from SCG to SPG. If the Pause pulse encoding is enabled by pulling EnPause signal high, the controller moves to PAUSE_PULSE state from the CRC_NIBBLE state. Otherwise, the current message sequence is accomplished, and the controller jumps back to SYNC_CALIB state for the next message sequence transmission. In the PAUSE_PULSE state, the controller waits for *N_pp_* clock ticks to ensure the total *N_ms_* cycles for fixed *T_ms_* message sequence duration according to Equations (4) and (5), respectively. The sensor information is encoded and transmitted as SENT message frames back-to-back continuously, until the IC is powered up, without any control signals from the ECU.

### 4.2. SENT CRC Cenerator (SCG)

The SENT CRC Generator is the key building block in the proposed design for computing the checksum for every 24-bit input Data nibbles. The CRC nibble is computed by using the polynomial of Equation (6) with initial 4-bit seed (0101)_2_:

(6) $$P\left(x\right)=\;x^{4}+x^{3}+x^{2}+1$$

**Figure 7:**
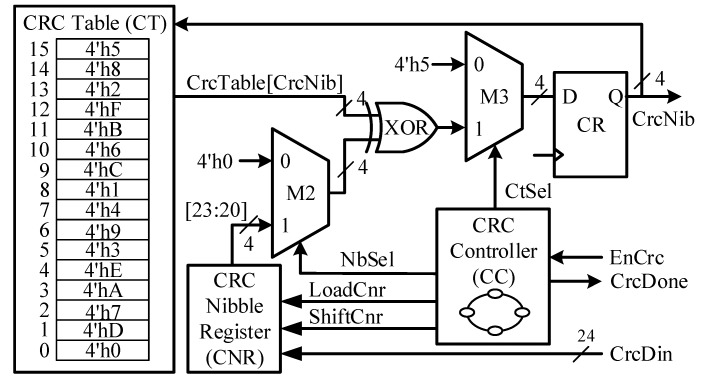
The SENT CRC generator architecture.

**Figure 8:**
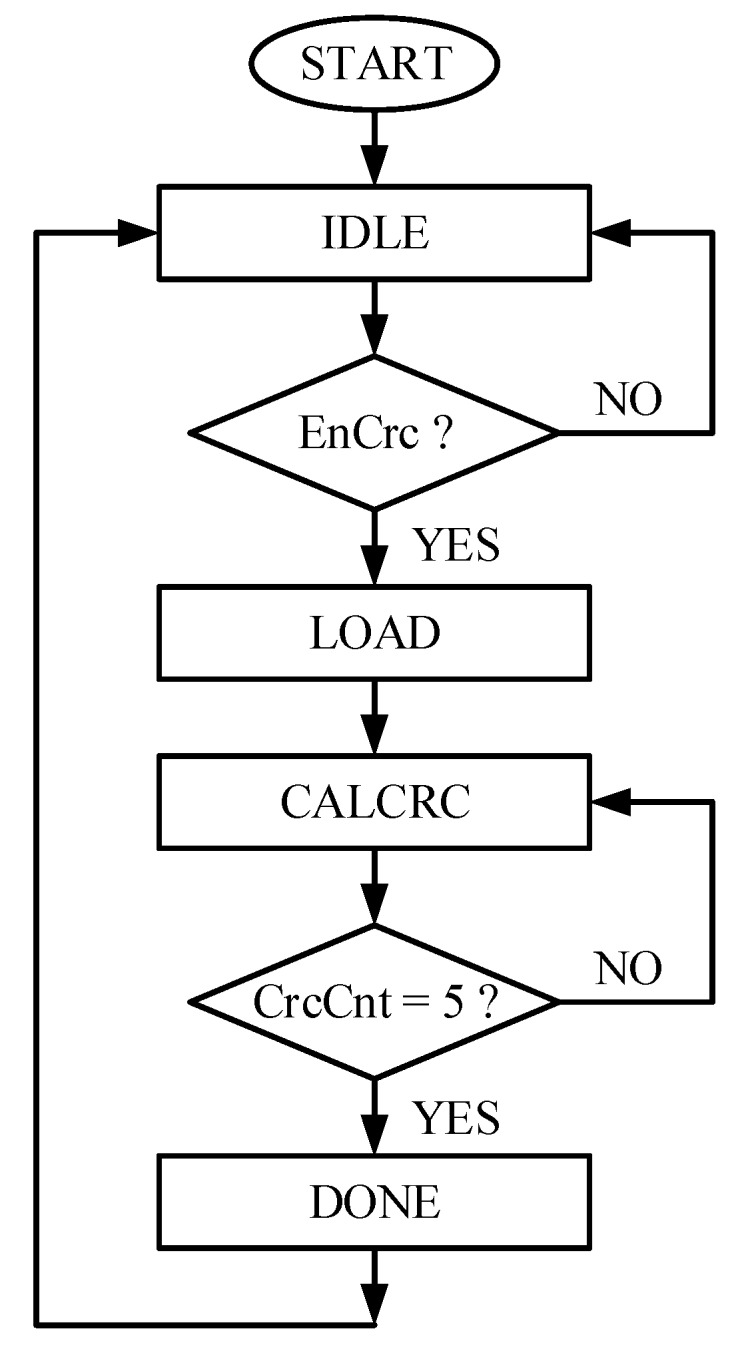
SENT CRC controller flow chart.

**Figure 9:**
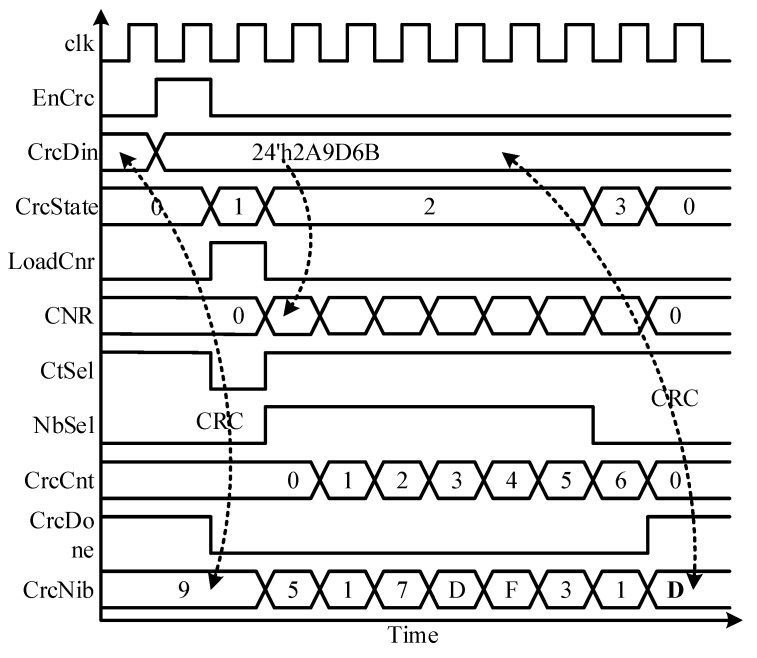
SENT CRC controller timing diagram.

Among the legacy and recommended CRC implementation types, defined in SAE J2716, later with 16-element array is adopted. This implementation requires very few memory elements for the CRC table, when compared with that of the 256-element array [17]. The CRC checksum is designed as a bitwise exclusive OR with a 16-element array lookup. The checksum is reckoned by using all data nibbles in sequence, and then check summing the result with an extra zero value. Figure 7 shows the architecture of the SCG block. It is mainly composed of the CRC controller (CC), CRC table (CT), CRC nibble register (CNR), CRC register (CR), and two multiplexers M2 and M3. The CNR is the 24-bit, parallel load, nibble shift left register, similar to NDR. The input sensor information nibbles are copied to this register for computing checksum. The CT is a 16-element lookup table, and it holds the unique, sequenced, and predefined 4-bit values [17], which are accessed by their addresses. The CRC controller follows the checksum algorithm and computes the final value for each message sequence. Figure 8 redraws the flow diagram of the CC, while Figure 9 plots the CNG timing diagram. When the SMC asserts the EnCrc signal high for one clock cycle, the CC moves to LOAD state from the IDLE state. The 24-input CrcDin is loaded to the CNR, and CR is set to its default value, by pulling the control signal CtSel low. On the next clock cycle, the checksum calculation starts in the CALCRC state. On every clock cycle, each data nibble is exclusive-ORed with one of the CT values, and stored in CR. The current CrcNib value acts as a read address for CT to access the respective value. When all the six nibbles are processed, the CC moves the DONE state for one clock cycle. The CRC nibble value is achieved by exclusive-ORing the zero with the final result of the previous state. The CrcDone asserts to high, and the controller jumps back into IDLE state. The CR keeps the final checksum value CrcNib during its sleep mode in this state. It takes eight clock cycles to compute the checksum for six nibbles of the current message sequence and remains in silent mode in IDLE state for the rest of time.

### 4.3. SENT Pulse Generator (SPG)

**Figure 10:**
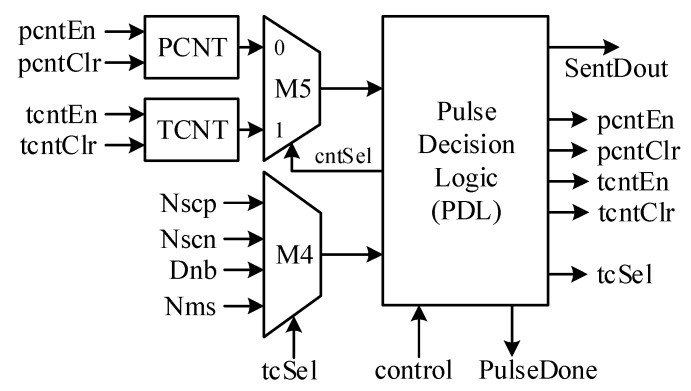
SENT pulse generator architecture.

The SENT Pulse Generator outputs all pulse types in predefined sequence controlled by the SMC. Figure 10 shows its architecture. When the Pause pulse is activated, the PCNT counter enables at the start of each message sequence and ensures constant message length equal to the *N_ms_* clock ticks. It resets to zero at the end of the frame when its value approaches *N_ms_*. To maintain the low and high widths of each pulse type, the clock tick counter, TCNT is incorporated. The pulse decision logic (PDL) generates the SENT pulse sequence based on the state of SMC. It controls, monitors, and compares the PCNT and TCNT values for their limits. The multiplexer M4 selects the upper values for counters. In SYNC_CALIB state, for first *N_low_* clock ticks, the dout remains low, and then pulls to high, until the TCNT hits the *N_scp_* value which is 56, as defined in [17]. Similarly, for STATUS_COMM state, the pulse width is *N_scn_* clock ticks, which is equal to *K_offset_* in this design. For DATA_NIBBLE and CRC_NIBBLE states, the value of each nibble *D_nb_* determines the pulse duration. In the PAUSE_PULSE state, the PCNT value is compared with *N_ms_* for constant message length for each frame. The output signal PulseDone asserts high for one clock at the end of the message sequence, to move the SMC back to SYNC_CALIB state, to encode the next frame.

### 4.4. Nibble Data Register (NDR)

**Figure 11:**
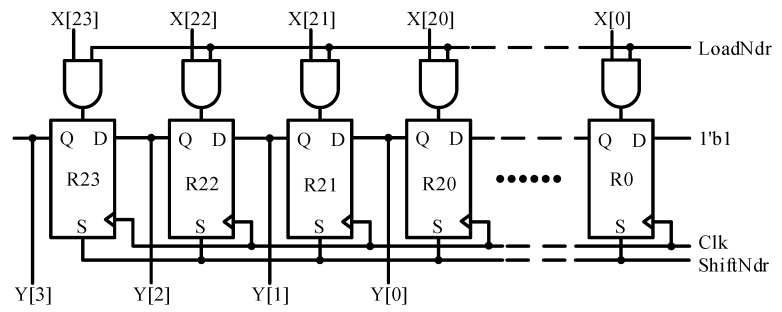
Nibble data register architecture.

Figure 11 shows the Nibble Data Register, which is a 24-bit parallel load shift register. The maximum possible six nibbles of sensor information from the DCM are sampled and loaded into this register for encoding. It holds their values until the transmission of the current message sequence is finished. During frame encoding, all the data at its input is ignored, and it samples only when the next message sequence starts. It shifts four bits to the left at a time, and the most significant nibble is directed to SPG through M1 multiplexer during each nibble encoding. The loading of 24-bit sensor information and then its shifting are governed by the SMC with LoadNdr and ShiftNdr control signals, respectively.

## 5. Reverse and over Voltage Protection (ROVP)

**Figure 12:**
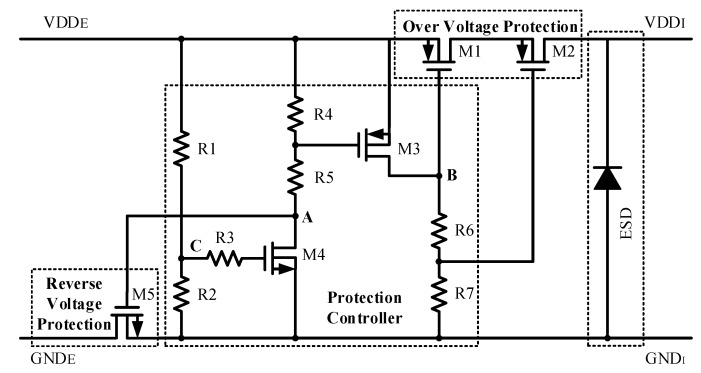
Reverse and over voltage protection with ESD circuit [21].

The reliability of electronics components in automotive applications is crucial. Therefore, to enhance the SENT transmitter reliability, and to make it compliant with AEC-Q100 [22], the highly reliable protection circuit is integrated with the proposed design to preserve the sensor signal conditioning IC from reverse and over voltages [21]. Figure 12 shows the circuit diagram of reverse and over voltage protection, which is composed of MOSFETs and resistors, instead of conventional comparator-based protection circuits. To prevent the transistors from breakdown, and to avoid the latch-up problem, the potential zeroing technique is adopted in reverse voltage protection mode.

The ESD is combined to protect the SENT transmitter from damage due to static electricity. Table 1 summarizes the behavior of transistors in different modes. In normal operation mode, the stacked transistors M1 and M2, with withstand voltages of 8 V are turned on, and VDD_EXT is transferred to VDD_INT with 29 mV voltage drop. In over voltage protection mode, these transistors are open to cut off the external voltage. When external voltage exceeds 8.2 V, M4 turns on by R1 and R2, M3 gate voltage forms due to R4 and R5, and it also turns on, and eventually M1 and M2 turn off by R6 and R7. The M4 turns the M5 off while both grounds are connected by M5 body diode. Due to the V_GS_ and V_DS_ of each transistor, the circuit is not damaged in the (−16 to 16) V range. In reverse mode, the external voltage drops below 0 V, and M4 is turned off, and node A between M4 and R5 follows this reverse voltage, which turns M5 off. In this way, both the grounds are isolated, and voltage between VDD_INT and GND_INT is 0 V, which protects the SENT transmitter in reverse voltage. The ESD diode is implanted to enhance the ESD performance and reliability of the protection circuit. In reverse mode, if both grounds are at 0 V, the PMOS body diode cannot endure reverse voltage. Therefore, for achieving 0 V between internal supply nodes, the potential zeroing approach is adopted.

## 6. Measurement and Simulation Results

**Figure 13:**
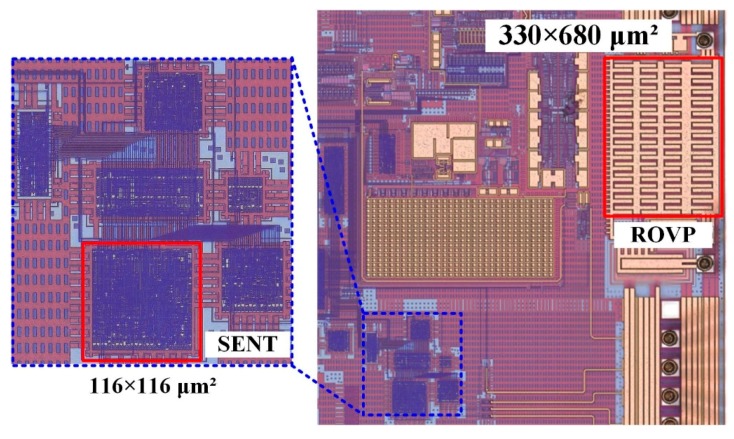
Chip microphotograph.

**Figure 14:**
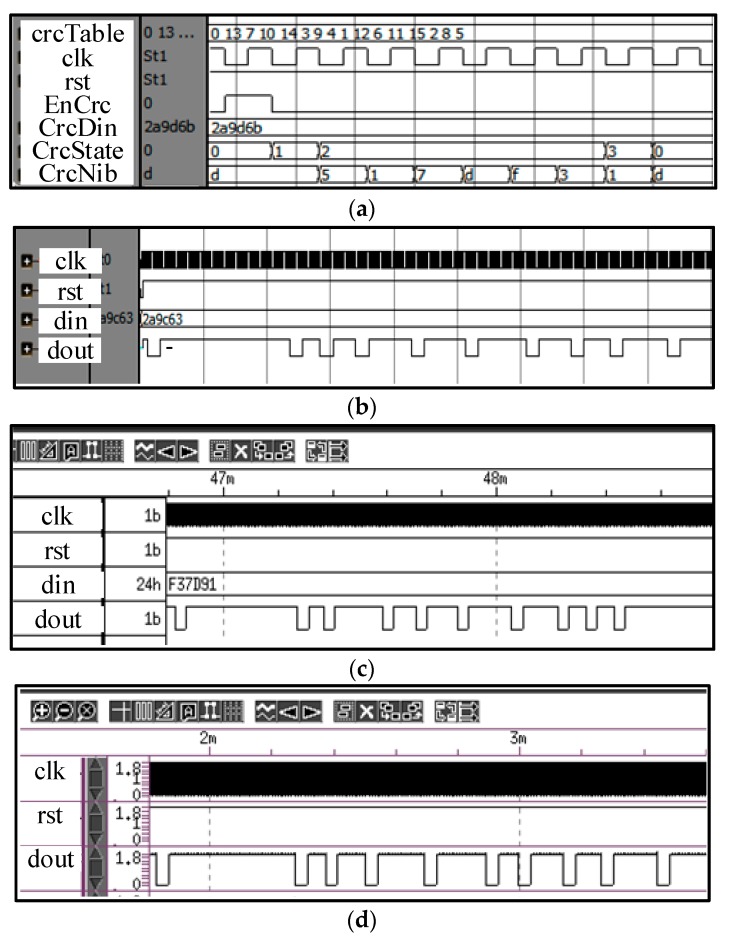
SENT simulation results. (**a**) CRC calculation with data (2A9D6B)_16_ and CRC (D)_16_; (**b**) Data (2A9C63)_16_, CRC (B)_16_, Clock Ticks 228, Duration 1.824 ms; (**c**) Data (F37D91)_16_, CRC (1)_16_, Clock Ticks 201, Duration 1.068 ms; (**d**) Data (4CD163)_16_, CRC (B)_16_, Clock Ticks 207, Duration 1.656 ms.

The presented SENT architecture is integrated with the pressure and temperature sensor signal conditioning IC for automotive applications as depicted in Figure 3. The microphotograph of the fabricated IC, marking the SENT and ROVP is captured in Figure 13. Table 2 summarizes the implementation characteristics of the design. This sensor IC is designed with 1P6M 180 nm CMOS process. The SENT layout occupies only 13.456 mm^2^ of total IC, and its implementation needs only 4.314 K gates. The power consumption of the architecture is very small, which is limited to 90 μW with 50 μA current from 1.8 V voltage source. The design is fully synthesizable and Verilog HDL is used to translate it into circuit level. The clock period is fixed to 8 μs, though it may be operated at much higher frequencies. Most of the SENT designs are implemented in software, microcontrollers or FPGA [25,26]. The SENT driver presented in [27] is implemented in 180 nm CMOS process and it occupies and an area of 0.9 mm × 0.7 mm. The 97.86% area reduction is achieved in the proposed SENT design. Figure 14 captures the different simulation results for the SENT module. Figure 14a shows the simulation of the CRC calculation. The checksum nibble (D)_16_ is computed from 24-bit input (2A9D6B)_16_. Figure 14b shows the functional simulation after HDL implementation, where the total message duration is 1.824 ms with 228 clock ticks for (2A9C63)_16_ six nibbles of data. Figure 14c,d gives snapshots of post place and route (P&R) and post layout simulation verification for (F37D91)_16_ and (4CD163)_16_ input test vectors with (201 and 207) clock ticks and (1.068 and 1.656) ms frame periods, respectively.

**Figure 15:**
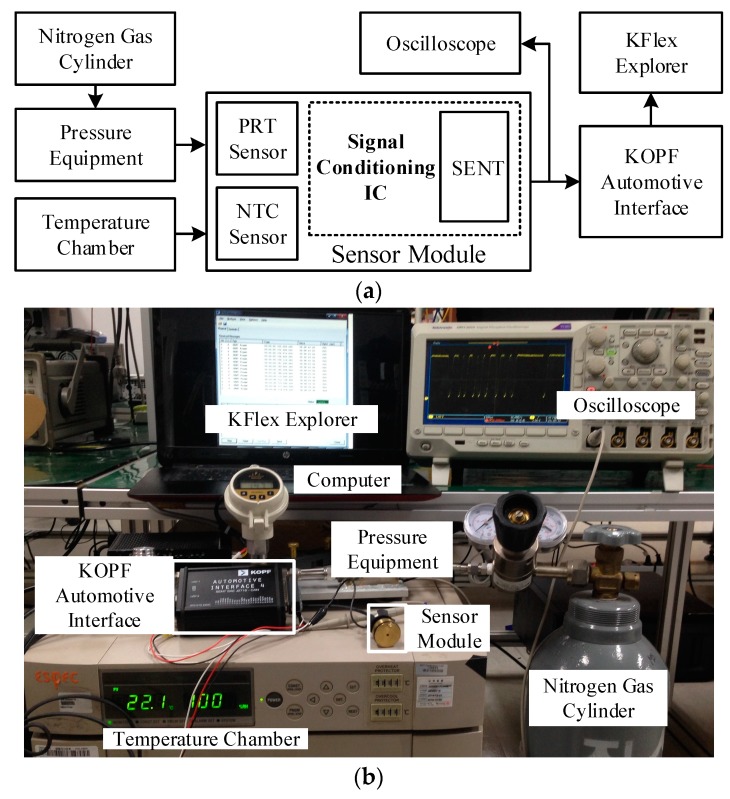
Measurement setup. (**a**) Measurement setup block diagram; (**b**) Measurement setup lab setup.

**Figure 16:**
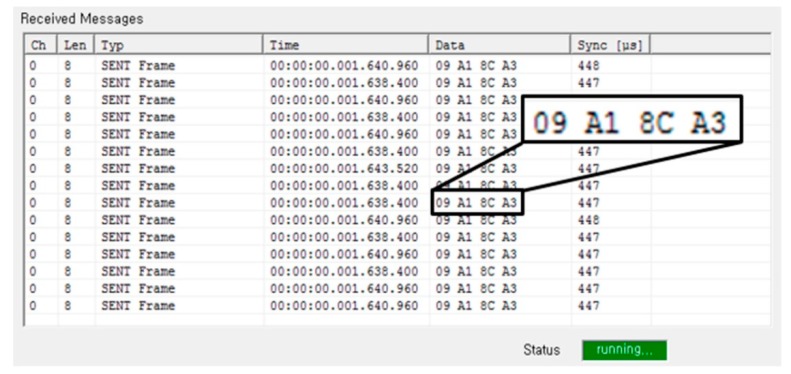
KFlexExplorer SENT message reception.

Figure 15 shows the experimental setup for measuring the proposed SENT design after the fabrication. Figure 15a gives the block diagram of the measurement framework, while Figure 15b shows the actual testing lab environment. For pressure measurement, the Sensor Module is fitted into Pressure Equipment, and then pressure is applied to the IC through PRT from the Nitrogen Gas Cylinder. Similarly, the Sensor Module is placed in the Temperature Chamber, and the IC is exposed to the temperature via NTC for temperature measurement, as is evident in Figure 15. After IC calibration, these quantities are encoded according to SENT message format. The KOPF Automotive Interface, a commercially available SENT monitor module compliant to SAE J2716 [28], receives the SENT traffic from the IC, and communicates it to KFlexExplorer for analysis. The KFlexExplorer is a Graphical User Interface (GUI) application running on Computer. It receives the SENT frames from KOPF Automotive Interface and analyzes these received messages according to the SENT standard [29]. Figure 16 provides a snapshot of KFlexExplorer during measurement.

Before measuring the pressure and temperature with the proposed SENTIC, it is first calibrated for the minimum and maximum input ranges for pressure and temperature. The resultant corresponding parameters, such as PGA gain, offset, linearity, minimum and maximum pressure, and temperature codes, etc. are saved in the IC internal memory block ROM. The digitally encoded measured values are mapped to actual values according the transfer function given in Equation (7).

(7) $$X_{c}=\;X_{min}+\;\frac{X_{max}-X_{min}}{Y_{max}-Y_{min}}\left(Y_{m}-Y_{min}\right)$$

**Figure 17:**
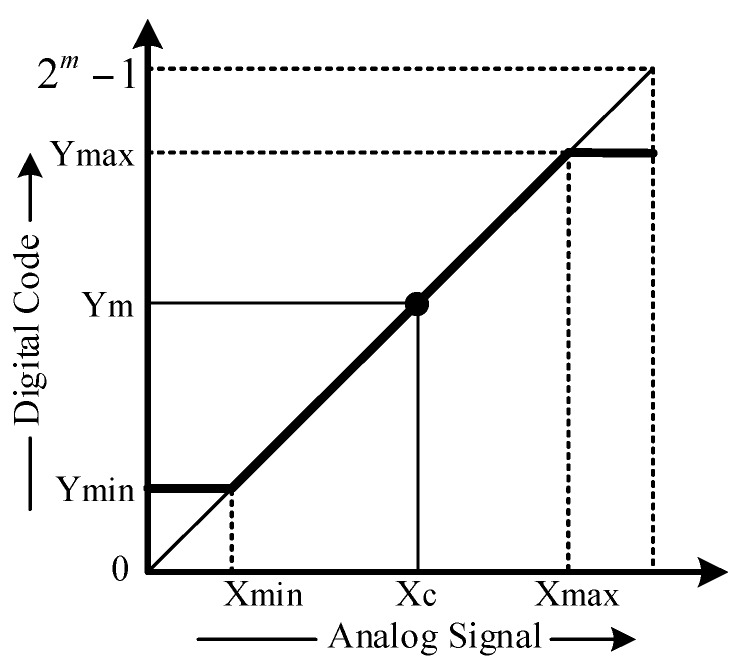
Transfer characteristics for measuring signal value.

Figure 17 shows the transfer characteristic curve for this equation, in which *Y_m_* is the measured digital code from the IC that is mapped to *X_c_* analog quantity. The *X_max_* and *X_min_* are the largest and smallest digital code values when maximum, *Y_max_* and minimum, *Y_min_* inputs are applied at the sensors, respectively. In the 24-bit SENT input signal, the 14 most significant bits, din<23:10> represent the pressure code, while the remaining ten least significant bits, din<9:0> hold the temperature information. Table 3 summarizes the measurable ranges for pressure and temperature and their corresponding digital codes during calibration.

**Figure 18:**
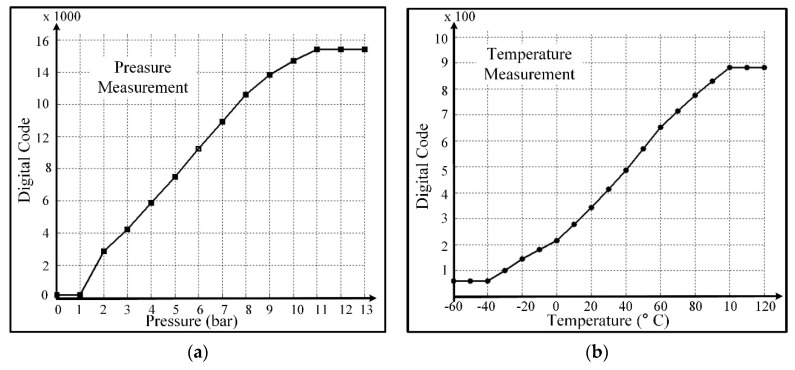
Pressure and temperature measurement with SENT. (**a**) Pressure measurement; (**b**) Temperature measurement.

**Figure 19:**
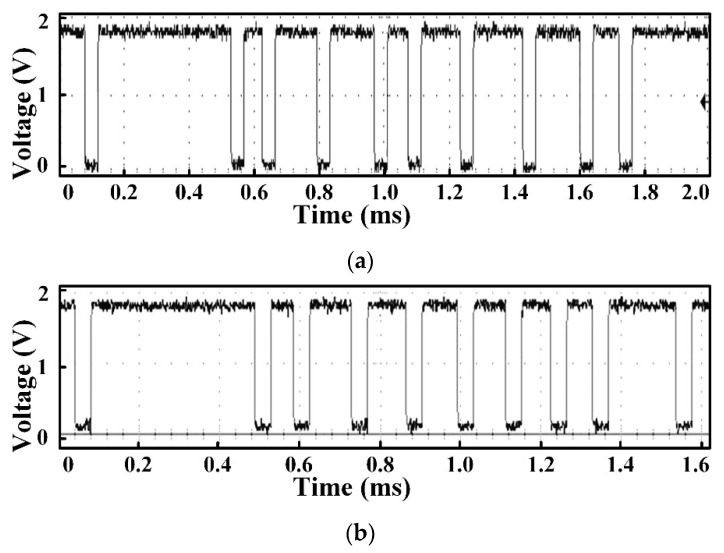
SENT measurement result. (**a**) Data (9A18CA)_16_, CRC (3)_16_, clock ticks 205, duration 1.640 ms; (**b**) Data (654321)_16_, CRC (E)_16_, clock ticks 187, duration 1.496 ms.

**Figure 20:**
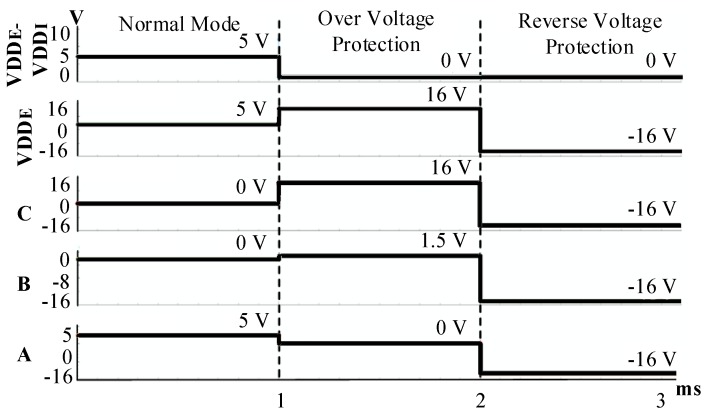
Reverse and over voltage protection simulation result.

**Figure 21:**
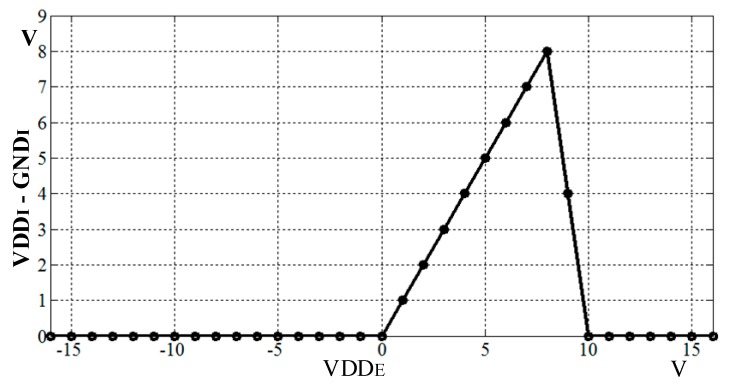
Reverse and over voltage protection measurement result.

In the measurement process, the pressure and temperature are applied on the IC separately, and Table 4 and Table 5 list the corresponding results, respectively. Figure 18 also plots the resulting data. The SENT digital codes are fixed to their respective range values when the applied pressure or temperature is beyond the calibrated limits. When applied pressure at the PRT sensor is increased from 1 bar to 11 bar, the SENT output also linearly increases from 149 to 15,436 respectively. If pressure is applied beyond 11 bar, the IC digital controller clamps its output, and hence the dout is fixed to 15,436, as clear from Figure 18a. The SENT design exhibits the same behavior for input temperature range, as explored in Figure 18b. After fabrication, the SENT protocol operation is verified extensively in different ways during IC measurements. In the digital controller MCB of the IC, there is a test pattern generator (TPG) module for SENT design Built-In Self-Test (BIST). In the test mode, TPG generates incremental patterns from (000000)_16_ to (FFFFFF)_16_ for SENT design verification. This BIST of SENT block is rigorously verified with KOPF Automotive Interface and KFlexExplorer. Furthermore, the dout pin of the IC is directly probed on the oscilloscope according to the measurement setup explained in Figure 15 for analyzing the encoding accuracy and timing features. The period of each pulse type is measured on the oscilloscope screen, and their corresponding type and value are decoded from Equations (1) and (2). The total message duration *T_ms_* is analyzed on oscilloscope, and then the total clock ticks *N_ms_* are calculated from Equation (5). Figure 19 illustrates the SENT message sequences encoded by the proposed SENT architecture. Figure 19a shows the 24-bit input data is (9A18CA)_16_ and calculated CRC value is (3)_16_. According to Equations (4) and (5), it takes 205 clock ticks, and is transmitted in 1.640 ms duration. When the transmission duration of this message sequence is measured on oscilloscope, it is also found to be 1.640 ms. Similarly, in Figure 19b, the measured and calculated results are the same, and it is found that the (654321)_16_, (E)_16_, 187, and 1.496 ms are the 24-bit data, 4-bit CRC, frame clock ticks, and the message period, respectively. All the simulation and measurement results ensure that the proposed SENT design is fully compliant with the protocol described in SAE J2716 standard. Figure 20 shows the protection circuit simulation results. It summarizes the results for reverse, over and normal modes. The node C is critical for both the over voltage protection mode, as well as normal mode. In normal mode, both nodes B and C are at 0 V, while node C is at 16 V, to block the over voltage in over voltage protection mode. The node A guides the reverse voltage protection mode by external voltage, VDD_E_. At code A, the potential zeroing is accomplished, and as a result, in normal operational condition, the protection circuit operates at 5 V with the protection range from (−16 to 16) V. Figure 21 shows the measurement results of the reverse and over voltage protection design. When the external voltage exceeds 8.2 V, VDD_I_ is blocked from the VDD_E_ by the protection circuit. The GND_I_ follows the reverse voltage, created by the protection controller in the reverse voltage protection operational mode. Figure 21 shows that the SENT transmitter is therefore protected. For reliable SENT implementation in automotive applications, the ESD related requirement is the following AEC-Q100 criteria [22]. The HBM criteria specified in AEC-Q100 is at least 2 kV, and an ESD is incorporated between VDD_I_ and GND_I_ to satisfy this reliability criterion in the protection circuit.

## 7. Conclusions

This paper has presented a low-power and small-area SENT transmitter design for automotive applications. The measurement and simulation results proved its full compliance with the SAE J2716 standard. The reverse and over voltage protection made it highly reliable and compliant to AEC-100Q for the harsh automotive environment. The fabrication of the suggested design used 1P6M 180 nm CMOS technology. The occupied area for SENT was very small, which was 13.456 mm^2^ and it had consumed only 4.314 K gates for its logic implementation. It consumed only 50 μA current from a voltage supply of 1.8 V, and thus limited the power requirement only up to 90 μW. After IC fabrication, SENT transmitter architecture was extensively tested with KOPF Automotive Interface and KFlexExplorer, and achieved 100% accuracy with zero error. This paper also included a reverse and over voltage design for the protection of SENT in highly reliable automotive applications. The architecture had a range of protection to over voltage of (8.2 to 16) V, and to reverse voltage of (−16 to 0) V. Also, in the ESD HBM test, the protection circuit satisfied the requirements at 6 kV. It occupied a 330 μm × 680 μm area. The low power consumption, small occupied area, and high operational accuracy of the proposed SENT transmitter design has made it very suitable for contemporary automotive applications. The design has proved its strength to be integrated with any automotive sensor to report sensor information to the ECU efficiently and accurately.

## Figures and Tables

**Table 1:** ROVP circuit operation summary.

Mode	M1	M2	M3	M4	M5
Normal	ON	ON	OFF	OFF	ON
Over protection	OFF	OFF	ON	ON	OFF
Reverse protection	OFF	OFF	OFF	OFF	OFF

**Table 2:** Design implementation summary.

SENT		ROVP	
Parameter	Value	Parameter	Value
Circuit	SENT transmitter	Circuit	Reverse and over protection circuit
Process	180 nm CMOS	Process	180 nm CMOS
Maximum current	50 μA	Integration level	On-chip
Supply voltage	1.8 V	Protection range	(−16–16) V
Power consumption	90 μW	ESD (HBM)	6 kV
Area	116 μm × 116 μm	Area	330 μm × 680 μm
Gate count	4.314 K		

**Table 3:** Pressure and temperature calibrated parameters.

Parameter	Pressure	Temperature
*m*	14	10
*Y_max_*	11 bar	100 °C
*Y_min_*	1 bar	−40 °C
*X_max_*	149	883
*X_min_*	15436	59

**Table 4:** Pressure measurement (bar).

Pressure	0	1	2	3	4	5	6
Code	149	149	2852	4232	5883	7493	9228
Pressure	7	8	9	10	11	12	13
Code	10,919	12,626	13,855	14,646	15,436	15,436	15,436

**Table 5:** Temperature measurement (°C).

Temperature	−60	−40	−30	−20	−10	0	10	20	30
Code	59	59	101	144	180	216	279	342	414
Temperature	40	50	60	70	80	90	100	110	120
Code	486	569	652	714	766	829	833	833	833
